# Clinico‐Genetic, Imaging and Molecular Delineation of *COQ8A* ‐Ataxia: A Multicenter Study of 59 Patients

**DOI:** 10.1002/ana.25751

**Published:** 2020-06-10

**Authors:** Andreas Traschütz, Tommaso Schirinzi, Lucia Laugwitz, Nathan H. Murray, Craig A. Bingman, Selina Reich, Jan Kern, Anna Heinzmann, Gessica Vasco, Enrico Bertini, Ginevra Zanni, Alexandra Durr, Stefania Magri, Franco Taroni, Alessandro Malandrini, Jonathan Baets, Peter de Jonghe, Willem de Ridder, Matthieu Bereau, Stephanie Demuth, Christos Ganos, A. Nazli Basak, Hasmet Hanagasi, Semra Hiz Kurul, Benjamin Bender, Ludger Schöls, Ute Grasshoff, Thomas Klopstock, Rita Horvath, Bart van de Warrenburg, Lydie Burglen, Christelle Rougeot, Claire Ewenczyk, Michel Koenig, Filippo M. Santorelli, Mathieu Anheim, Renato P. Munhoz, Tobias Haack, Felix Distelmaier, David J. Pagliarini, Hélène Puccio, Matthis Synofzik

**Affiliations:** ^1^ Department of Neurodegenerative Diseases, Hertie‐Institute for Clinical Brain Research and Center of Neurology University of Tübingen Tübingen Germany; ^2^ German Center for Neurodegenerative Diseases (DZNE) University of Tübingen Tübingen Germany; ^3^ Neurorehabilitation Unit, Department of Neurosciences IRCCS Bambino Gesù Children Hospital Rome Italy; ^4^ Department of Systems Medicine University of Roma Tor Vergata Rome Italy; ^5^ Institute of Medical Genetics and Applied Genomics University of Tübingen Tübingen Germany; ^6^ Department of Pediatric Neurology University Children’s Hospital Tübingen Germany; ^7^ Morgridge Institute for Research Madison WI USA; ^8^ Department of Biochemistry University of Wisconsin‐Madison Madison WI USA; ^9^ Brain and Spine Institute (ICM) Sorbonne Université, Pitié‐Salpêtrière University Hospital Paris France; ^10^ AP‐HP, Department of Genetics Pitié‐Salpêtrière University Hospital Paris France; ^11^ Unit of Neuromuscular and Neurodegenerative Diseases, Department of Neurosciences Bambino Gesù Children’s Hospital, IRCCS Rome Italy; ^12^ Unit of Medical Genetics and Neurogenetics Fondazione IRCCS Istituto Neurologico Carlo Besta Milan Italy; ^13^ Department of Medicine, Surgery, and Neurosciences University of Siena, Unit of Neurology and Neurometabolic Disorders, Azienda Ospedaliera Universitaria Senese Siena Italy; ^14^ Neurogenetics Group, University of Antwerp Antwerp Belgium; ^15^ Institute Born‐Bunge University of Antwerp Antwerp Belgium; ^16^ Department of Neurology Antwerp University Hospital Antwerp Belgium; ^17^ Service de Neurologie, Université de Franche‐Comté, CHRU de Besançon Besançon France; ^18^ Unité Extrapyramidale, Département des Neurosciences Cliniques HUG, Faculté de Médecine, Université de Genève Geneva Switzerland; ^19^ Praxis für Humangenetik Erfurt Erfurt Germany; ^20^ Department of Neurology Charité University Medicine Berlin Berlin Germany; ^21^ Suna and Inan Kıraç Foundation, Neurodegeneration Research Laboratory KUTTAM, Koç University School of Medicine Istanbul Turkey; ^22^ Behavioural Neurology and Movement Disorders Unit, Department of Neurology Istanbul Faculty of Medicine, Istanbul University Istanbul Turkey; ^23^ Departments of Pediatric Neurology Dokuz Eylül University Faculty of Medicine İzmir Turkey; ^24^ Department of Diagnostic and Interventional Neuroradiology University of Tübingen Tübingen Germany; ^25^ Department of Neurology, Friedrich‐Baur‐Institute Ludwig‐Maximilians University of Munich Munich Germany; ^26^ German Center for Neurodegenerative Diseases (DZNE) Munich Germany; ^27^ Munich Cluster for Systems Neurology (SyNergy) Munich Germany; ^28^ Department of Clinical Neurosciences University of Cambridge Cambridge UK; ^29^ Institute of Genetic Medicine Newcastle University Newcastle UK; ^30^ Department of Neurology Radboud University Medical Centre, Donders Institute for Brain, Cognition and Behaviour Nijmegen The Netherlands; ^31^ Centre de Référence Maladies Rares “Malformations et Maladies Congénitales du Cervelet” Paris‐Lyon‐Lille France; ^32^ Département de Génétique et Embryologie Médicale APHP, GHUEP, Hôpital Armand Trousseau Paris France; ^33^ Developmental Brain Disorders Laboratory Imagine Institute, INSERM UMR 1163 Paris France; ^34^ Hôpital Femme Mère Enfant Service de Neuropédiatrie Bron France; ^35^ Hôpitaux universitaires Pitié Salpêtrière ‐ Charles Foix, Service de Génétique Paris France; ^36^ EA7402 Institut Universitaire de Recherche Clinique, and Laboratoire de Génétique Moléculaire CHU and Université de Montpellier Montpellier France; ^37^ IRCCS Fondazione Stella Maris Pisa Italy; ^38^ Service de Neurologie, Hôpitaux Universitaires de Strasbourg Hôpital de Hautepierre Strasbourg France; ^39^ Fédération de Médecine Translationnelle de Strasbourg (FMTS) Université de Strasbourg Strasbourg France; ^40^ Institut de Génétique et de Biologie Moléculaire et Cellulaire (IGBMC) INSERM‐U964/CNRS‐UMR7104/Université de Strasbourg Illkirch France; ^41^ Movement Disorders Centre, Toronto Western Hospital University of Toronto, Krembil Research Institute Toronto Ontario Canada; ^42^ Department of General Pediatrics, Neonatology, and Pediatric Cardiology University Children's Hospital Duesseldorf, Medical Faculty, Heinrich Heine University Duesseldorf Germany; ^43^ Institut de Génétique et de Biologie Moléculaire et Cellulaire (IGBMC) Illkirch France; ^44^ INSERM, U1258 Illkirch France; ^45^ CNRS, UMR7104 IIllkirch France; ^46^ Université de Strasbourg Strasbourg France

## Abstract

**Objective:**

To foster trial‐readiness of coenzyme Q8A (COQ8A)‐ataxia, we map the clinicogenetic, molecular, and neuroimaging spectrum of COQ8A‐ataxia in a large worldwide cohort, and provide first progression data, including treatment response to coenzyme Q10 (CoQ10).

**Methods:**

Cross‐modal analysis of a multicenter cohort of 59 COQ8A patients, including genotype–phenotype correlations, 3D‐protein modeling, in vitro mutation analyses, magnetic resonance imaging (MRI) markers, disease progression, and CoQ10 response data.

**Results:**

Fifty‐nine patients (39 novel) with 44 pathogenic *COQ8A* variants (18 novel) were identified. Missense variants demonstrated a pleiotropic range of detrimental effects upon protein modeling and in vitro analysis of purified variants. COQ8A‐ataxia presented as variable multisystemic, early‐onset cerebellar ataxia, with complicating features ranging from epilepsy (32%) and cognitive impairment (49%) to exercise intolerance (25%) and hyperkinetic movement disorders (41%), including dystonia and myoclonus as presenting symptoms. Multisystemic involvement was more prevalent in missense than biallelic loss‐of‐function variants (82–93% vs 53%; *p* = 0.029). Cerebellar atrophy was universal on MRI (100%), with cerebral atrophy or dentate and pontine T2 hyperintensities observed in 28%. Cross‐sectional (n = 34) and longitudinal (n = 7) assessments consistently indicated mild‐to‐moderate progression of ataxia (SARA: 0.45/year). CoQ10 treatment led to improvement by clinical report in 14 of 30 patients, and by quantitative longitudinal assessments in 8 of 11 patients (SARA: −0.81/year). Explorative sample size calculations indicate that ≥48 patients per arm may suffice to demonstrate efficacy for interventions that reduce progression by 50%.

**Interpretation:**

This study provides a deeper understanding of the disease, and paves the way toward large‐scale natural history studies and treatment trials in COQ8A‐ataxia. **ANN NEUROL 2020;88:251–263**

Next‐generation sequencing techniques have recently unraveled a large number of novel autosomal‐recessive cerebellar ataxia (ARCA) genes, providing an increasing share of previously undiagnosed patients with ataxia with a molecular diagnosis.^1^ This genetic progress now needs to be translated into preparation of first treatment trials, prioritizing in particular those genetically stratified ARCAs where targeted treatment options might be within reach.^1^ ARCA due to mutations in coenzyme Q8A (*COQ8A*; formerly: *ADCK3*; now also referred to as ATX‐COQ8A^2^) might serve as a particularly promising candidate disease, as its underlying molecular pathogenesis includes aberrant coenzyme Q10 (CoQ10) synthesis, which might be susceptible to drug treatments, eg, CoQ10 supplementation.^3^ Although smaller case series described first experiences with CoQ10 in COQ8A‐ataxia,^4^, ^5^, ^6^, ^7^ the design and conduction of treatment trials in COQ8A‐ataxia is—as paradigmatic for almost all ARCAs—hampered by lack of systematic knowledge on the full phenotypic spectrum, effects of different gene variants, and disease progression data.

To overcome these limitations and to take the first steps toward trial readiness, we here leverage a worldwide multicenter cohort of 59 patients with biallelic *COQ8A* variants to characterize the phenotypic, molecular, and neuroimaging spectrum of the disease. This includes structural modeling and molecular in vitro analysis of *COQ8A* variants, magnetic resonance imaging (MRI) biomarker candidates, diffusion tensor imaging, and first quantitative progression data, including response to CoQ10 treatment and preliminary sample size calculations for future treatment trials.

## Methods

### Patients and Deep‐Phenotyping Assessment

Patients with rare biallelic variants in *COQ8A* were compiled from ataxia centers from 15 different sites in Europe and North America. Patients or records were systematically assessed by the local physician according to a common comprehensive data sheet, including prespecified queries about genetics, demographics, history, phenotype, disease severity, and treatment experience as well as muscle histology, laboratory, electrophysiology, and MRI findings (Appendix S1). Twenty previously published reports on patients^5^, ^6^, ^8^, ^9^ were systematically re‐assessed in‐depth for this study. All patients had at least one neurological examination; 25 patients had ≥2 prospective examinations. Disease severity was assessed by the Scale for the Assessment and Rating of Ataxia (SARA),^10^ and the Spinocerebellar Degeneration Functional Score (SDFS).^11^ All patients or legal representatives provided informed consent according to local regulations.

### Variant Selection

*COQ8A* variants were identified by different sequencing methods, depending on local facilities and year of sampling, either by high throughput panel or exome sequencing or—more rarely—by direct sequencing of *COQ8A*. All *COQ8A* variants were annotated according to the Ensembl variant effect predictor (VEP) in January 2019.^12^ Loss of function (LOF) variants (frameshift, stop, and splice) were included if minor allele frequency (MAF) was <1% in the Genome Aggregation Database (gnomAD). Frameshift variants, stop variants, and splice variants at canonical splice sites were considered likely pathogenic; splice variants outside canonical splice sites as variants of unknown significance (VUS). Missense variants were considered to be likely pathogenic if they met all of the following criteria: (i) MAF <1%, (ii) combined annotation dependent depletion (CADD) score (phred‐like, version 1.4) >20,^13^ and (iii) at least 2 of 3 in silico prediction scores predicted pathogenicity, specifically SIFT <0.05, PolyPhen 2 > 0.5 or fathmm/MKL > 0.5.^14^, ^15^, ^16^ Missense variants were included as VUS if they met all of the following criteria: (i) MAF <1%, (ii) CADD score 10–20, and (iii) at least 1 of 3 in silico prediction scores (see above) predicted pathogenicity. Segregation of the variants with disease was assessed in family members, whenever DNA was available.

### Structural Modeling and in vitro Analyses of *COQ8A* Variants

Structural modeling predictions were built on in silico mutagenesis tools PyMOL and Coot, based on the published COQ8A crystal structures (PDB ID: 4ped and 5i35).^17^, ^18^ In addition, five representative recurrent *COQ8A* variants with different clinical phenotypes and in different protein domains were selected for further comparative in‐depth molecular analysis: three variants at motifs of the COQ8A active site (R271C near the KxGQ motif, A338T in A‐rich loop, and T487R at catalytic loop), one variant at a site not presumed to participate in catalysis (R301W in GQα3), and one VUS (D326N). Recombinant wildtype (WT) and selected mutant COQ8A^NΔ250^ protein (truncated N‐terminal targeting sequence and transmembrane domain) were expressed in *E. coli* and purified for in vitro analysis of ATPase activity, protein stability, and nucleotide binding capacity, as described before.^17^, ^18^, ^19^ A catalytically inactive D507N mutant (COQ8A activation loop) and a catalytically hyperactive A339G mutant (A‐rich loop) served as controls. A malachite green based ATPase activity assay is based on the activation of COQ8A^NΔ250^ by 2‐propylphenol (2‐PP).^19^ Protein stability was determined by differential scanning fluorimetry.^20^ Stabilization of COQ8A^NΔ250^ by the addition of adenosine diphosphate (ADP) in this assay indicates nucleotide binding capacity.

### Magnetic Resonance Imaging

Findings from routine brain MRIs were systematically aggregated from all patients where such MRI results were available. In addition, digital routine brain MRIs, including T1‐weighted, T2‐weighted, diffusion‐weighted images, and fluid attenuated inversion recovery T2 (FLAIR) images were systematically assessed by two central independent raters (B.B. and A.T.), where such images were available and digitally transferable for centralized review. For in‐depth analysis of fiber tracts, diffusion tensor imaging (DTI; 32 directions, b = 1,000 mm^2^/sec, 2 mm^3^ isotropic resolution) was performed in 3 patients and 12 matched healthy controls (4 men and 8 women; mean age 22 years; 19–24 years) with an Spin Echo‐Echo Planar (SE‐EPI) sequence on a 3 T scanner (Skyra; Siemens, Erlangen, Germany). After standard preprocessing with FSL and normalization with FNIRT, z‐Maps were generated by subtracting the mean FA image of all controls from each patient and afterward a division by the SD of the FA of all controls.

### Statistical Analysis

All statistics were calculated using GraphPad Prism 5 (GraphPad Software, La Jolla, CA) and SPSS 25 (IBM Corp., Armonk, NY). Data‐driven clusters of phenotypic features within the spectrum of COQ8A‐ataxia were searched by a statistical two‐step cluster analysis, taking the most prevalent features (ataxia, cognitive impairment, epilepsy, myoclonus/dystonia, and exercise intolerance) as input. For analyzing clinicogenetic associations, genotype was stratified (i) by the number of LOF alleles (two vs one vs zero), and (ii) by the presence of missense variants in specific target motifs (at least one missense variant in target motif vs all other patients with at least one missense variant). Clinicogenetic associations were then analyzed by testing for significant associations between these genotypic groups and (i) a phenotype of “ataxia simplex” (patient cluster with ataxia without evidence of the other four most prevalent features), or (ii) recurrent non‐ataxia features (>10% prevalence to reliably assume an intrinsic feature of COQ8A rather than an unrelated coincidence). To obtain robust results, we restricted our comparisons only to subgroups which comprise >5 patients per group (thus including only AAAS, GQa1, and GQa3), combined with use of the Fisher’s exact test as a robust and conservative test for small sample sizes to avoid accumulation of alpha errors.

Kaplan–Meier estimation was used to assess occurrence of ataxia and dependence on walking aids and wheelchair. Cross‐sectional annual disease progression was estimated by the ratio of each subject’s ataxia severity (last SARA score) and ataxia duration, as established previously.^21^, ^22^ Longitudinal annualized disease progression was determined by the slope of a linear regression through SARA scores across all available assessment intervals *within each individual*, after normalization to baseline age and score, assuming a linear progression of the SARA score in the course of ataxia.^23^ Based on these intraindividual disease progression slopes, we then calculated the mean progression across the whole group. Sample size calculations (two‐tailed *t*‐test, alpha = 0.05, power = 0.8) were based on the mean and the variability (SD) of the annualized change in SARA score during specified assessment intervals, and performed using G*Power 3.1.9.2^24^ after confirming normality distribution by the Shapiro–Wilk test.

## Results

### Mutational Landscape of *COQ8A*

**FIGURE 1:**
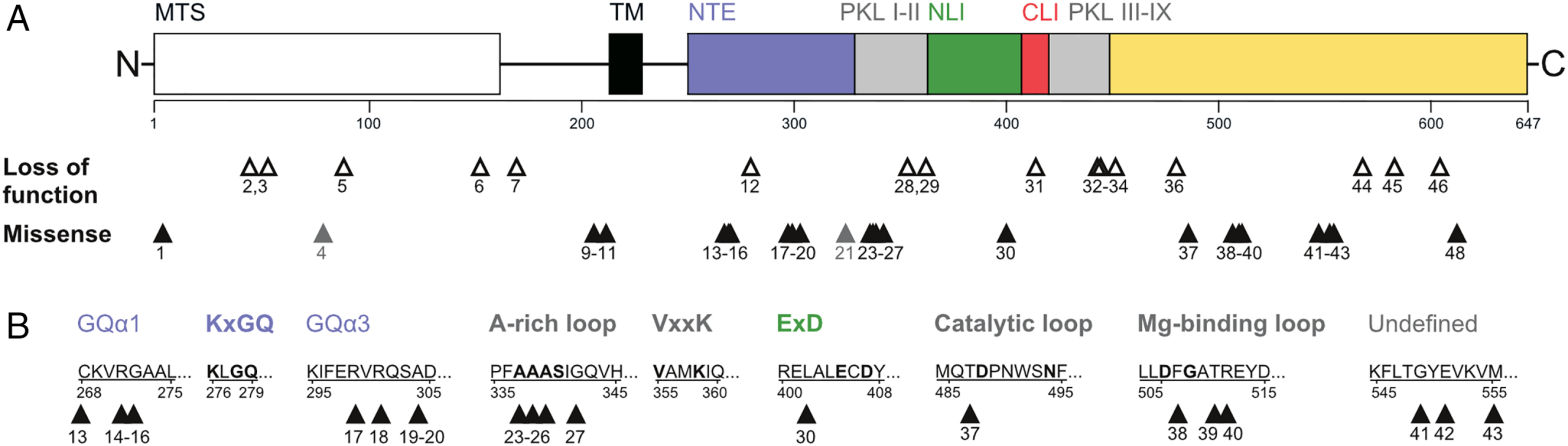
Coenzyme COQ8A variants across protein domains. (A) Graphical overview of all variants in this study in relation to COQ8A protein structure and domains, including the mitochondrial targeting sequence (MTS), the transmembrane domain (TM), subdomains of the protein kinase‐like (PKL) and superfamily (PKL I‐IX), as well as the UbiB family‐specific N‐terminal extension (NTE), N lobe insert (NLI), and C lobe insert (CLI). Numbers indicate variant IDs specified in the Table S1, missense variants of unknown significance are marked in gray. (B) Missense variants in relation to specific sequence motifs. Bold titles and letters indicate functional motifs and conserved amino acids of the COQ8A active site, respectively. Variants cluster predominantly near structural or functional motifs, except for one cluster affecting a yet undefined region. [Color figure can be viewed at www.annalsofneurology.org]

We identified 64 patients from 51 families with biallelic variants in *COQ8A*. Based on our genetic curation criteria, 59 patients, 39 of whom never reported before, carried 2 likely pathogenic variants, whereas 5 patients carried VUS on one or both alleles. Patients originated from 16 different countries (Appendix S1). Twenty six patients came from multiplex families with two affected siblings. Twenty one patients from 14 families had known consanguineous parents. We identified 44 different likely pathogenic variants (including 18 novel), comprising of 26 missense and 18 LOF (10 frameshift, 4 stop, 3 canonical splice, and 1 in‐frame deletion) variants (Table S1). Although spread across almost the entire *COQ8A* gene (Fig 1A), many missense variants clustered at functionally defined motifs: one at mitochondrial target sequence (MTS), three at transmembrane domain (TM), four at the KxGQ motif, five at AAAS motif of A‐rich loop, one at the ExD motif, one at the catalytic loop, and three at the Mg‐binding DFG motif of the activation loop (Fig 1B). Except for the AAAS motif, these key motifs were invariably hit *near* but not directly within these motifs. Recurrent missense variants were also found as clusters in two regions not presumed to participate in catalysis (4 between amino acids 299 and 304 in GQα3, and 3 between 549 and 555; see Fig 1B).

In an additional four patients (family #48–50), we identified three *COQ8A* variants meeting our criteria for VUS. This includes the variant c.993C>T (F331=), which has previously been published as pathogenic based on moderate skipping of exon 8 and reduced CoQ10 levels in lymphoblasts,^3^ but which is likely benign given an MAF of 1.6%.

### Variant Effects on COQ8A Protein Structure

**FIGURE 2:**
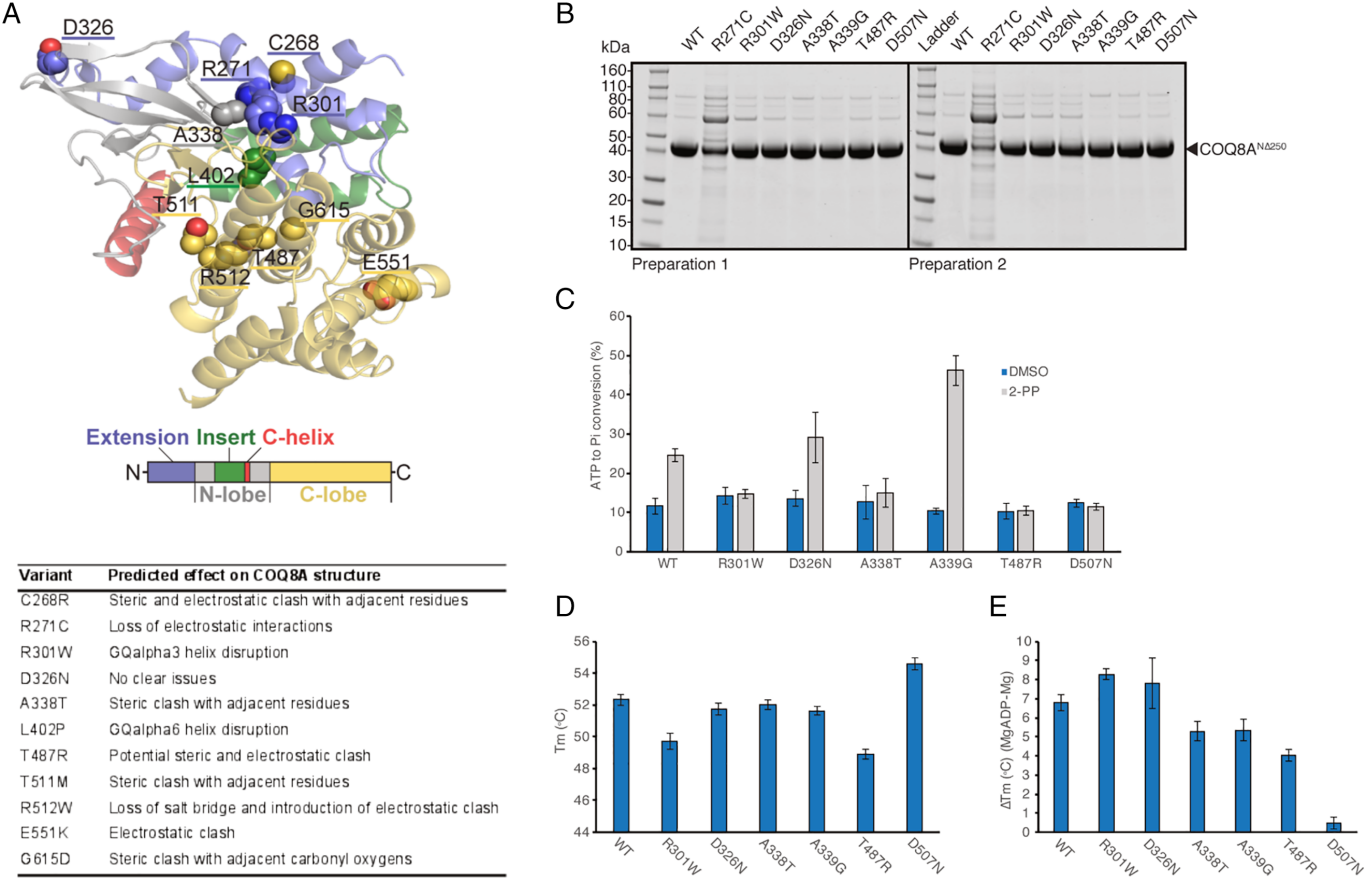
Molecular effects of representative coenzyme COQ8A variants. (A) The 3D modeling of protein structure and function, revealing steric and electrostatic clashes or lost interactions for representative *COQ8A* variants. Missense variants cluster around the active size of COQ8A, except for three variants including E551K. (B) SDS‐PAGE analysis of purified wildtype (WT) and mutant COQ8A^NΔ250^. Protein stability and folding was severely impaired in R271C, leading to purification failure in two preparations. (C) Compared to WT, ATPase activity was impaired in variants affecting presumed noncatalytic (R301W) and catalytic regions (A338T and T487R) of the active site. Positive control: A339G, and negative control: D507N. (D) Decrease in melting temperature (Tm) of purified *COQ8A* indicating moderate protein destabilization in R301W and T487R. (E) ADP nucleotide binding, which increases Tm, is impaired in A338T and T487R, but not in R301W. Note that variants of uncertain significance (VUS) D326N is not predicted to lead to detrimental structural effects A and consistently yields results similar to WT in all experiments B to E. [Color figure can be viewed at www.annalsofneurology.org]

We next assessed the predicted effects of all 48 variants on COQ8A protein structure, leveraging 3D structural modeling (Appendix S2; illustration of 11 selected variants in Fig 2A). All frameshift and stop variants led to termination of the protein, each eliminating essential regions of COQ8A. Missense variants predominantly grouped around the active site of COQ8A, except for variants clustering at position 549 to 555. The missense variants identified in our study were mainly predicted to cause (i) steric and/or (ii) electrostatic clashes, (iii) transmembrane or GQα helix disruption, (iv) loss of amino acid interactions, or (iv) mitochondrial targeting impairments. Variants at position 268 to 272 and 299 to 304 reciprocally disrupt interaction between GQα1 and GQα3, suggesting a common functional cluster. For the VUS D326 and Y607 as well as the likely benign variant F331=, no detrimental effects were predicted, although Y607 may lead to truncation of COQ8A by a cryptic splice site. VUS H80Y might impact mitochondrial targeting.

### In Vitro Analyses of Selected *COQ8A* Variants

Five selected *COQ8A* variants of different phenotypes, protein positions, and assumed pathogenicity were further analyzed in vitro in an *E. coli* expression model, including effects on ATPase activity, protein stability, and nucleotide binding. All variants could be purified, except R271C, which only co‐purified with a likely chaperone contaminant, thus indicating its detrimental effect on protein stability and folding (Fig 2B). This exceptional in vitro finding, which predicts this variant to be highly disruptive, corresponds to the severe phenotype of the patient with this homozygous variant (P31): the patient presented with an extraordinarily severe, early‐onset multisystemic phenotype with severe developmental delay, and never learned to walk. Out of the other variants, ATPase activity was impaired in *COQ8A* mutants A338T (P1 and P13) and T487R (P5 and P18) affecting the active site of the protein, but also in R301W (P2, P21, P22, P24, and P41) at a site not presumed to participate in catalysis (Fig 2C), thus adding further functional support for the pathogenicity of these variants. In contrast, the VUS D326N (P22) exhibited WT‐like ATPase activation, adding support that it is likely not pathogenic. This corresponds with the atypical clinical phenotype of the patient carrying this variant in a homozygous state (P51): the phenotype included—in contrast to all 59 patients with likely pathogenic *COQ8A* variants—optic atrophy and predominant sensory ataxia. *COQ8A* variants R301W and T487R also exhibited moderate protein destabilization (Fig 2D). Finally, nucleotide binding capacity was impaired for the disruption of the COQ8A active site by variants A338T and T487R, but not by R301W (Fig 2E).

### Phenotypic Features of COQ8A‐Ataxia

**FIGURE 3:**
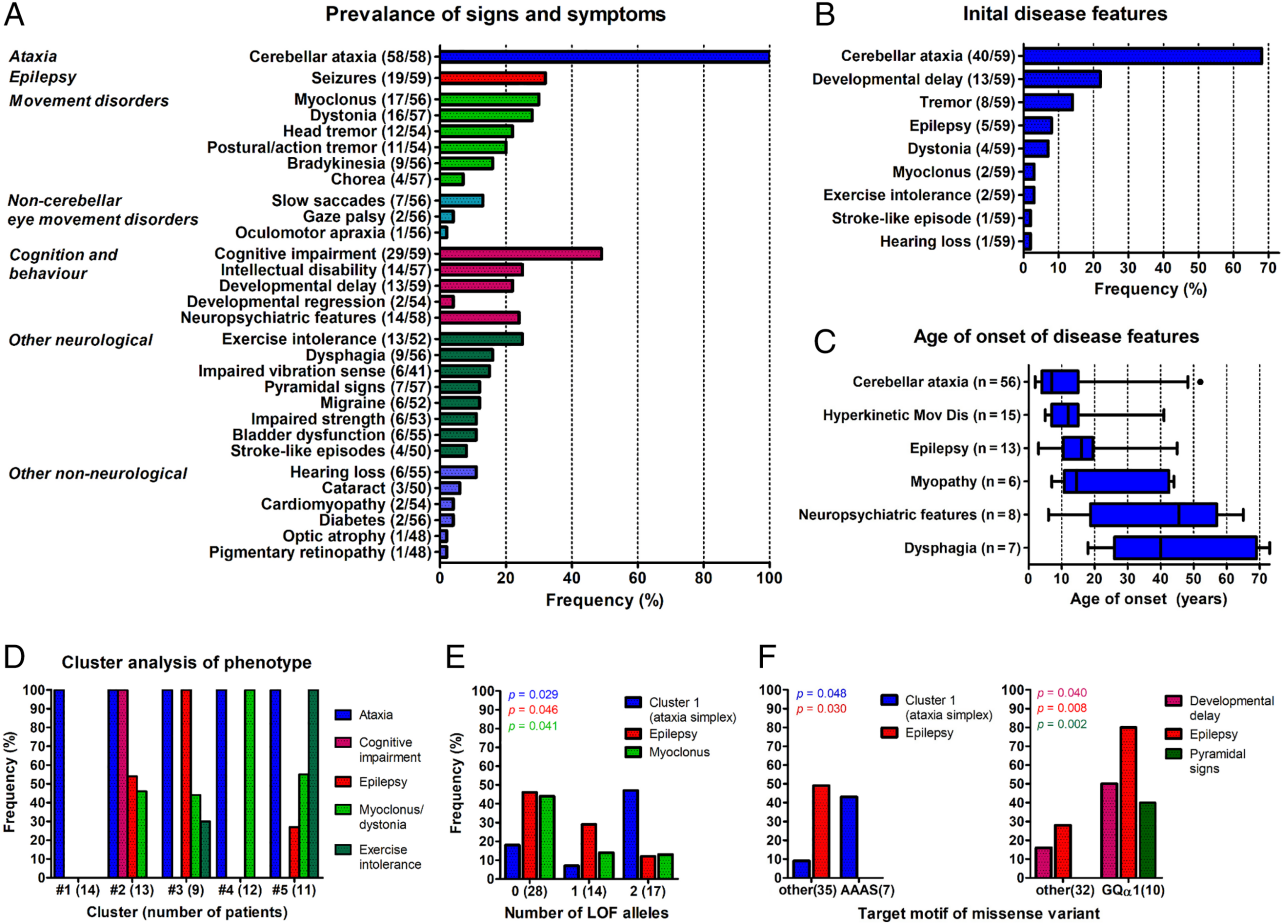
Phenotypic spectrum of coenzyme COQ8A‐ataxia. (A) Prevalence of signs and symptoms in patients with *COQ8A* mutations (n = 59). Numerator and denominator in brackets indicate the number of affected patients and the number of patients assessed for this feature, respectively. (B) Frequency of initial features at disease onset. Thirteen of 59 patients had more than one feature at onset. Note that cerebellar ataxia was only present in 68% at disease onset. (C) Age of onset of disease features. Number of affected patients in brackets. Box‐whisker plots representing median, interquartile range, and 95% confidence interval (CI). (D) Frequency of the most prevalent features in five clusters identified by a two‐step cluster analysis. Note cluster 1 comprising of 14 patients (24%) with a cerebellar ataxia phenotype, but none of the other 4 prevalent features (“ataxia simplex” cluster), and cluster 4 comprising of 12 patients (20%) with ataxia and only myoclonus or dystonia. (E, F) Genotype–phenotype associations. Frequency of clinical features stratified by the number of loss of function (LOF) alleles (E), and by clusters of missense variants in specific COQ8A motifs (F). The *p* values represent results from Fisher’s exact tests. [Color figure can be viewed at www.annalsofneurology.org]

Clinical data were systematically analyzed across all the 59 patients with 2 likely pathogenic *COQ8A* variants (last examination: median 23 years after disease onset, range 3–68). Cerebellar ataxia, present in all patients (100%; see Fig 3A for the prevalence of all signs and symptoms), was the initial feature of the disease in 68% of patients (Fig 3B, C), with a median onset of 7 years (interquartile range [IQR] 4–15 years). Hyperkinetic movement disorders were observed in 41% of patients, including myoclonus (30%), dystonia (28%), head tremor (22%), and postural/action tremor (20%; see Fig 3A). Notably, myoclonus, dystonia, and prominent tremor were the initial presenting disease features in two, four, and eight patients, respectively, including two patients with *pure* dystonia at onset (P1 and P8). Dystonia manifested as cervical dystonia or focal dystonia of the upper limb in all patients. Other prevalent features were: cognitive impairment in 49% of patients (associated with intellectual disability in about 48% of these cases), epilepsy in 32%, and myopathic features (exercise intolerance or muscle weakness) as well as neuropsychiatric symptoms (depression, anxiety, psychotic symptoms, or impulsive‐aggressive behavior), each in about 25% of patients (see Fig 3A). Epilepsy and myopathic symptoms commonly started at juvenile age, whereas neuropsychiatric symptoms mostly evolved in later disease stages. Hearing loss (n = 6), stroke‐like episodes (n = 4), cataract (n = 3), diabetes (n = 2), or optic atrophy (n = 1), which are often considered as “classical” clinical signs of mitochondrial disease, occurred in a minority of patients (see Fig 3A).

### Genotype–Phenotype Associations in COQ8A‐Ataxia

To identify possible phenotypic clusters within this pleiotropy of COQ8A‐associated phenotypic features, a cluster analysis was performed using the most prevalent phenotypic features—epilepsy, myoclonus/dystonia, cognitive impairment, and exercise intolerance—as input variables. A two‐step cluster analysis revealed five phenotypic clusters with good cohesion and separation (silhouette coefficient: 0.7), including the existence of a cluster of 14 patients (24%) with cerebellar ataxia but none of the other four most prevalent features (=“ataxia simplex” cluster), and a cluster of 12 patients (20%) with ataxia and only myoclonus or dystonia (Fig 3D; see Appendix S3 for comparison of the subject subcohorts constituting each cluster).

In an exploratory analysis, we next analyzed whether the “ataxia simplex” cluster or individual non‐ataxia clinical features, which have been found to be prevalent (ie, at least 10%) in our COQ8A cohort, might be associated with certain COQ8A mutation types or affected protein domains (Appendices S4 and S5). The ataxia simplex cluster was more frequent in patients with biallelic LOF variants than in patients with missense variants (biallelic LOF: 47%, biallelic missense: 18%, Fisher’s exact test, *p* = 0.029). This indicates, in turn, that missense variants are more associated with multisystemic damage beyond ataxia (LOF: 53% and missense: 82–93%; Fig 3E). Correspondingly, epilepsy (biallelic missense: 46%, and biallelic LOF: 12%; *p* = 0.046) and myoclonus (biallelic missense: 44%, and biallelic LOF: 13%; *p* = 0.041) were more prevalent in patients with biallelic missense mutations than in patients with LOF variants. The pattern of more prevalent ataxia simplex (43 vs 9%; *p* = 0.048) and absent epilepsy (49 vs 0%; *p* = 0.030) was just found for missense variants in the AAAS motif, suggesting that these mutations—the only ones to hit a functional COQ8A motif directly—may be functionally equivalent to LOF mutations (Fig 3F). Compared to all other missense variants, variants affecting helix GQα1 in the KxGQ domain were more frequently associated with developmental delay (50 vs 16%; *p* = 0.040), epilepsy (80 vs 28%; *p* = 0.008), and pyramidal signs (40 vs 0%; *p* = 0.002).

### Routine and DTI MRI Brain Imaging

**FIGURE 4:**
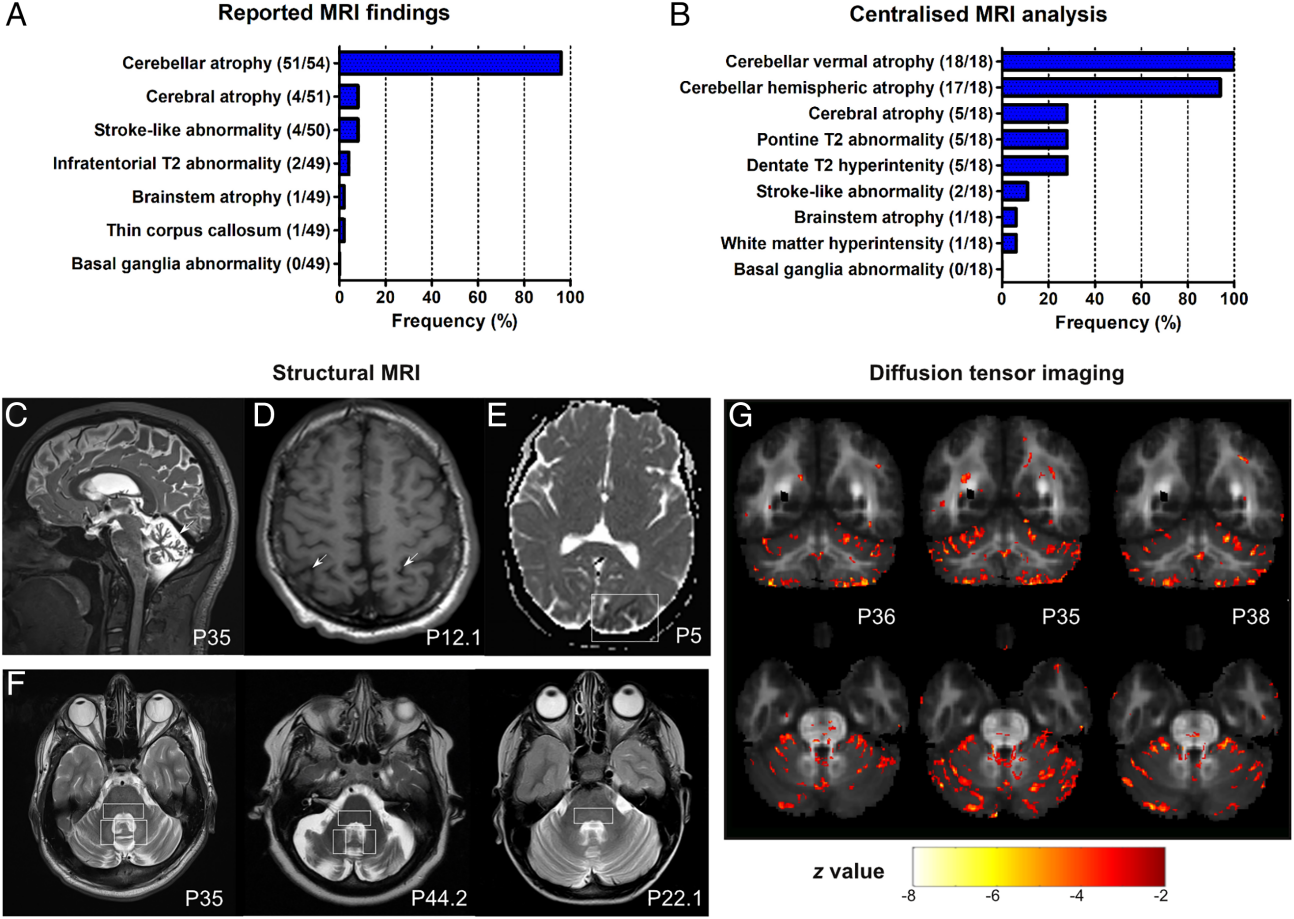
Magnetic resonance imaging (MRI) features of coenzyme COQ8A‐ataxia. (A) Reported MRI findings. Numerator and denominator in brackets indicate the number of patients with a feature and the number of patients assessed for this feature, respectively. (B) Centralized analysis of original MRI images of 18 patients by 2 independent raters. Vermal cerebellar atrophy was universally present. Representative images highlighting cerebellar atrophy (C), cerebral atrophy (D), and stroke‐like abnormalities (E), and infratentorial T2 hyperintensities of the dorsal pons, and dentate nuclei (F) as a novel imaging features of COQ8‐ataxia. (G) Diffusion tensor imaging (DTI) analysis. The z‐map of three individual COQ8A patients (P36, P35, and P38) superimposed on the mean FA map of all controls, depicting voxels with a z‐score less than −2.5 and a cluster size of 30. In all three patients, FA values are reduced in the periphery of the anterior and posterior lobe, the superior cerebellar peduncle and pontine crossing tracts, and diffusely in supratentorial clusters. [Color figure can be viewed at www.annalsofneurology.org]

MRI analyses were based on (i) reported findings (available for 54 of 59 patients; Fig 4A), complemented by a (ii) centralized in‐depth re‐assessment of digital brain MRIs (available for 18 of 59 patients, mean disease duration at MRI: 15.9 years, range: 1–64) by two independent raters (Fig 4B; see Appendix S6 for comparison of subcohorts available for imaging analyses). The reported findings described cerebellar atrophy as an almost universal finding (94% of patients; except for three patients between 9 and 25 years of age) and less frequently cerebral atrophy (8%), stroke‐like abnormalities (8%), infratentorial signal abnormalities (4%), and brainstem atrophy (2%; Fig 4D–F). The centralized in‐depth rating of the images revealed vermal atrophy in 100% (18 of 18), atrophy of the cerebellar hemispheres in 94% (17 of 18; Fig 4C), and cerebral atrophy in 28% (5 of 18), with a parietal and fronto‐insular predominance in 3 of them (3 of 5). Infratentorial signal abnormalities were also re‐identified in 28% (5 of 18; visible across 3 different scanners), consisting of hyperintense dentate nuclei and a hyperintensity of the dorsal pons on T2 images (see Fig 4F). In contrast to the relatively high prevalence of hyperkinetic movement disorders observed in our cohort, no structural basal ganglia abnormalities were observed in these routine MRIs. Standardized DTI in three patients (P36, P35, and P38) unraveled changes of infratentorial fiber tracts in all three patients, specifically reduced FA values in the periphery of the anterior and posterior cerebellar lobe, as well as in the superior cerebellar peduncle and pontine crossing tracts, accompanied by mild and disseminated FA changes across supratentorial fiber tracts (Fig 4G).

### Evolution and Progression of COQ8A‐Ataxia

**FIGURE 5:**
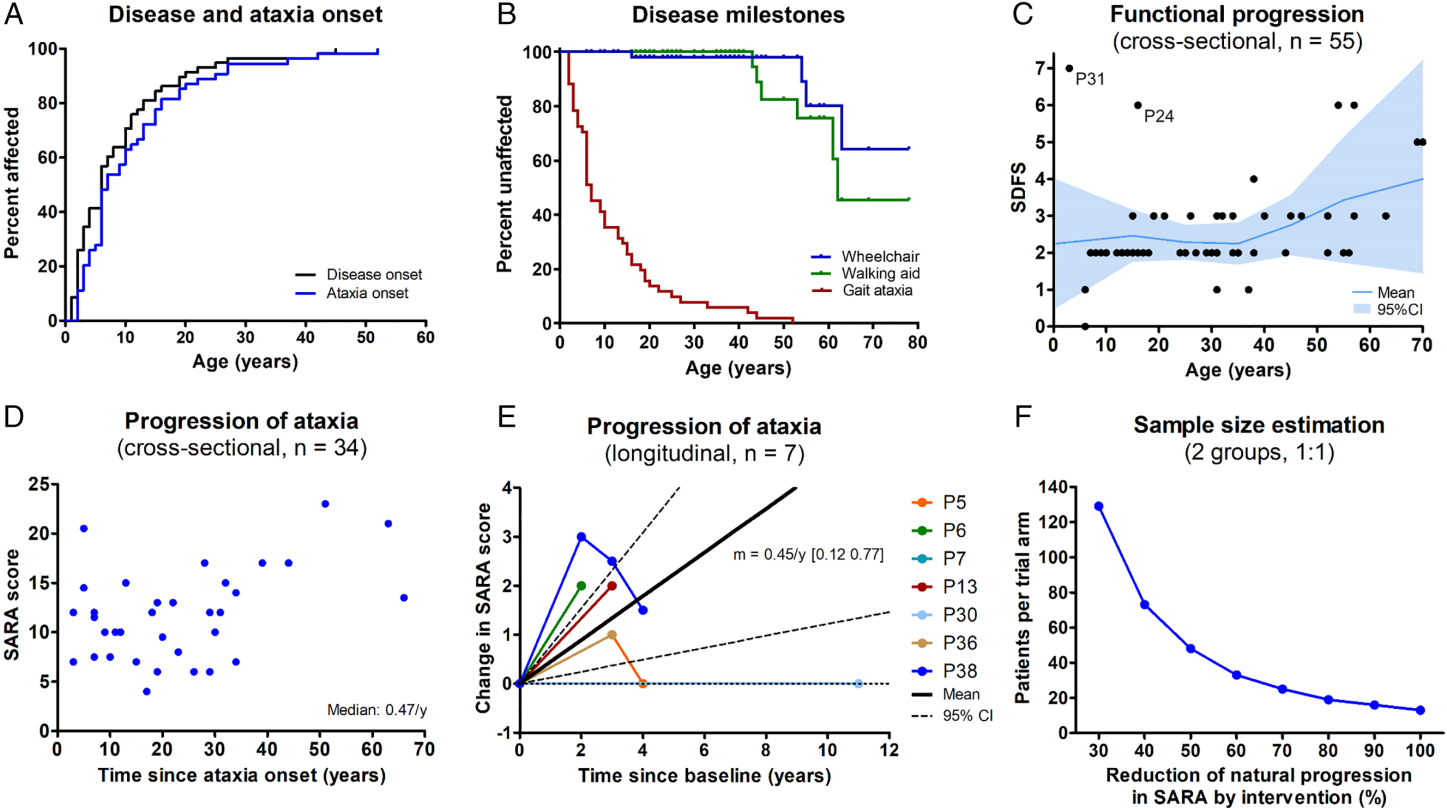
Disease progression of coenzyme COQ8A‐ataxia. (A) Both overall disease and ataxia start early in life in COQ8A‐ataxia, with 50% of patients affected before age 6 years. (B) Kaplan–Meier analysis of disease milestones. Because of the limited number of subjects aged >60 years, estimates above age 60 years should be interpreted with caution. (C) Cross‐sectional functional disease progression as indicated by the Spinocerebellar Degeneration Functional Score (SDFS) relative to age. SDFS 3 reflects moderate ataxia with the inability to run, and SDFS 6 reflects wheelchair dependence. Mean and 95% confidence interval based on 10‐year bins. (D) Cross‐sectional ataxia progression as indicated by the individual Scale for the Assessment and Rating of Ataxia (SARA) score at the last assessment relative to disease duration. (E) Prospective longitudinal progression of ataxia. Relative progression of each patient quantified by the slope of a linear regression through all available SARA scores. Solid and dashed lines indicate mean and 95% confidence interval. Note that variation in disease course/SARA ratings can appear as “improvements” even in the natural course of COQ8A disease. (F) Sample size estimation based on longitudinal progression of SARA scores during the first assessment interval (0.59 ± 0.51 per year) for a trial to detect a treatment effect with a power of 0.8 and a significance level of 0.05. [Color figure can be viewed at www.annalsofneurology.org]

Disease onset occurred before 6 years of age in 50% of patients, and all patients (100%) developed disease symptoms before 45 years of age (Fig 5A). Dependence on a walking aid or wheelchair were estimated to occur in about 55% (95% confidence interval [CI]: 26–88%) and 35% (95% CI: 13–76%) of patients, respectively, but not before 40 and 50 to 60 years of age, respectively (Kaplan–Meier estimate; Fig 5B), indicating an on average relatively mild to moderate overall disease progression. This was also reflected by the cross‐sectional SDFS scores, indicating mild (SDFS 2) to moderate (SDFS 3) ataxia in 82% of patients (47 of 57), and dependence on two walking aids (SDFS 5) or a wheelchair (SDFS 6) only in 11% (6 of 57) of patients, most of them above 50 to 60 years of age (Fig 5C). However, observations from single subjects indicate that COQ8A‐ataxia can also follow a much worse disease trajectory in specific individuals, partly even including premature death at juvenile age (see Fig 5C): P31 (homozygous R271C mutation; age 5 years at last examination) never learned to walk due to severe motor and cognitive retardation and eventually developed drug‐resistant epilepsy and spastic tetraparesis, and P24 (homozygous R301W mutation; age 16 years at last examination), who was bedridden due to intractable seizures and subsequently died at age 17 because of severe dilated cardiomyopathy.

Cross‐sectional ataxia progression, estimated by the cross‐sectional ratio of ataxia severity (SARA score; available for 34 patients) and disease duration, indicated a median ataxia progression rate of 0.47 SARA points per year (Fig 5D; see Appendix S7 for comparison of subcohorts available for progression estimates). Prospective longitudinal disease progression data (available for 7 drug‐naïve patients) revealed a mean annualized change of 0.45 SARA points per year (95% CI: 0.12–0.77), thus corroborating the cross‐sectional estimate, yet also indicating some variation in the SARA scores appearing as “improvements” in the natural disease course (Fig 5E). The annualized change in SARA score during the first assessment interval of each patient (n = 7; 0.59 ± 0.51 per year) was used to calculate an exploratory estimate of the minimally required sample size to detect effects of a hypothetical intervention (Fig 5F). With this preliminary approach, at least 48 patients per trial arm would be required to detect a 50% reduction in annual SARA progression.

### Response to CoQ10 Treatment

#### Response to CoQ10 Treatment by Clinical Reports

**FIGURE 6:**
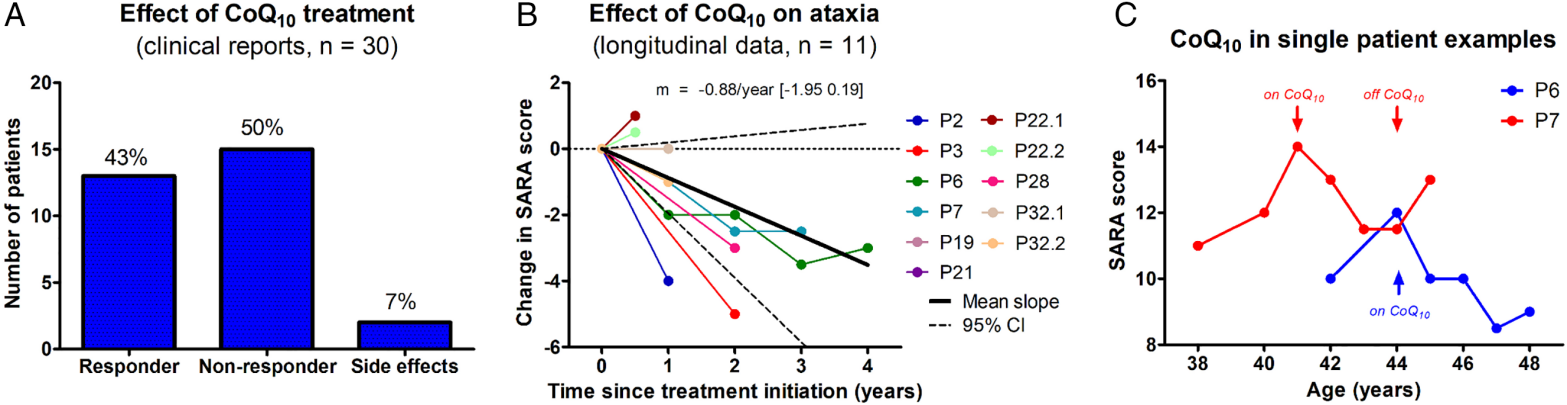
Effect of coenzyme Q10 (CoQ10) treatment. (A) Treatment response by clinical reports, suggesting improvements under CoQ10 in 43% of patients. (B) Treatment response by longitudinal assessment. Longitudinal changes in individual subjects’ Scale for the Assessment and Rating of Ataxia (SARA) scores (slope of a linear regression through all available SARA scores) after initiation of CoQ10 treatment. Solid and dashed lines indicate mean and 95% confidence interval of annualized change in SARA. (C) Trajectory of ataxia severity in two exemplary individual patients with longitudinal SARA scores both on and off treatment with CoQ10. A similar rate of each deterioration and improvement can be seen in these two patients, with a possible rebound after discontinuation of treatment in patient P6. [Color figure can be viewed at www.annalsofneurology.org]

Thirty patients (51%) were treated with CoQ10 supplementation, with a mean cumulative daily dose of 11 mg/kg/day (range: 2–40). Based on the clinical reports, 13 of 30 patients (43%) were classified as responders, and 15 of 30 (50%) as nonresponders (Fig 6A). Side effects (anorexia or diarrhea) were reported in 2 of 30 patients (7%). Qualitative descriptions of treatment effect were illustrative in that not only improvement of ataxia, but also of tremor, dystonia, epilepsy, mental speed, and/or muscle weakness were reported. Functional disease severity was lower in responders than nonresponders (SDFS: 2.3 [range: 1–6] vs 3.4 [range: 2–7], Mann–Whitney U test, *p* = 0.012). No associations were observed between treatment response and age of disease onset, age and disease duration at treatment initiation, ataxia severity (SARA score), cumulative daily dose, mutation type, or selected clinical, imaging, or laboratory features (Appendix S8). Of note, improvement was also reported in two patients without CoQ10 deficiency in muscle tissue.

#### Response to CoQ10 Treatment by Quantitative Longitudinal Assessment

Quantitative longitudinal treatment assessment of ataxia with the SARA score was available in a subgroup of 11 patients. Annual change in SARA score across all assessment intervals showed an average improvement of −0.88 points per year (95% CI: −1.95 to 0.19) under CoQ10 treatment (Fig 6B). In two patients (P6 and P7) with longitudinal assessments both with and without CoQ10 treatment, SARA scores deteriorated by about one point per year before treatment and after discontinuation of treatment, whereas it improved sequentially by about one point per year under CoQ10 treatment (Fig 6C).

## Discussion

This study leverages the largest worldwide cohort of COQ8A patients reported so far to facilitate first steps toward trial‐readiness of COQ8A‐ataxia. It provides a systematic characterization of the full phenotypic spectrum, the effects of a large range of *COQ8A* variants, and disease progression data, including response to CoQ10 treatment.

### COQ8A‐Ataxia: A Variable Phenotypic Spectrum, Including Dystonia Onset

Our findings characterize COQ8A‐disease as a variable multisystemic disease, with early‐onset cerebellar ataxia as a common denominator—either as the initial presenting symptom or manifesting later during the disease course. Corroborating and extending previous smaller case series, we demonstrate that it is mostly complicated by cognitive deficits, hyperkinetic movement disorders, epilepsy, or myopathic symptoms.^3^, ^4^, ^6^, ^8^ However, an “ataxia simplex” cluster also exists in 25% patients who do not show any of these particular additional signs. Focal dystonia can be an initial feature of COQ8A‐ataxia,^7^ even *preceding* any cerebellar ataxia. With a prevalence of 30%, dystonia seems to be a key feature in COQ8A‐disease, exceeding estimates from previous smaller case series.^5^, ^6^, ^8^ Ataxia with only dystonia or myoclonus even presents a partly distinct phenotypic cluster within the spectrum of COQ8A‐disease. Although cognitive deficits have been noted as mental retardation in some children with COQ8A‐ataxia, but were not systematically noted in adults with COQ8A‐ataxia.^3^, ^8^ Our study revealed cognitive deficits in up to 50% of patients, including later cognitive decline independent of pre‐existing intellectual disability in about half of them.

### Genotype–Phenotype Associations in COQ8A‐Ataxia

Our findings provide preliminary indications that multisystemic phenotypes, and, in particular, epilepsy and myoclonus, in COQ8A‐ataxia might be more frequently associated with missense variants than LOF variants, suggesting a possible gain‐of‐function or dominant‐negative mechanism for missense variants.^25^ This hypothesis warrants further specific work‐up in experimental models of COQ8A missense variants. In addition, our findings indicate that missense variants in helix GQα1 in the UbiB protein‐specific KxGQ domain might be associated with clinical signs reflecting cortical (developmental delay and epilepsy) and pyramidal tract dysfunction. With the exception of three missense variants in the AAAS motif (associated with “ataxia simplex” similar to LOF variants), no missense variant directly hit conserved amino acids of key functional motifs of the COQ8A active site, giving rise to the speculation that variants in these particular motifs might be associated with different phenotypes or even early lethal outcomes.

However, beyond these preliminary associations, no specific genotype–phenotype associations seem to exist. This might be because, as shown here by structural 3D protein modeling and in vitro analysis, different COQ8A missense variants act via a large diversity of mechanisms, including differential effects on ATPase activity, protein stability, and/or nucleotide binding capacity. In addition, our observation that even the same *COQ8A* mutation can present with different phenotypic features (eg, ataxia and hyperkinetic movement disorders in P20 vs ataxia and epilepsy in P23, both carrying the homozygous R348Ter mutation) and disease course (eg, disease onset age 11 years in P26.1 vs 1 year in P26.2 and P40, all with the homozygous M555I mutation) might be explained by differences in heteroplasmic mitochondrial DNA pools and additional cis‐ and trans‐acting genetic modifiers, which can differentially affect mitochondrial‐nuclear genome interactions, CoQ synthesis, and mitochondrial functioning.^26^ Moreover, also environmental and lifestyle factors, such as socioeconomic status, physical activity, or other health behaviors might contribute to the different phenotypic expression. Given the pleiotropic phenotype and wide range of effects of *COQ8A* variants, a thorough functional work‐up of newly identified variants is needed, as even ultra‐rare and well‐conserved *COQ8A* variants might not be pathogenic, as demonstrated here (eg, D236N and Y607=). Such analyses prevent premature inferences on putative novel phenotypes (as tempting, eg, for patient P22) and erroneous inclusion in upcoming treatment trials.

### MRI Imaging Reveals Widespread Infra‐ and Supratentorial Brain Damage, Including Dentate and Pontine Hyperintensities

The systematic analysis of the comprehensive MRI aggregation highlights cerebellar atrophy as the predominant MRI feature in COQ8A‐ataxia, corroborating earlier small case series.^3^, ^4^, ^6^, ^8^, ^27^ Cerebellar atrophy can be limited to the vermis, and, thus, be overlooked in particular in young patients at early stages of the disease (patients P2, P10.1, and P19). Cerebellar fiber changes include the periphery of the anterior and posterior cerebellar lobe, the superior cerebellar peduncle, and pontine crossing tracts, thus indicating a widespread cerebellar damage in COQ8A‐ataxia. Corresponding to the multisystemic clinical phenotype, underlying brain damage in COQ8A‐disease is not limited to the cerebellum, but also includes the brainstem and supratentorial regions, with parietal und fronto‐insular cerebral atrophy in >25% of patients, as shown here for the first time. The identification of T2 hyperintensities of the dentate nuclei and the dorsal pons present novel recurrent imaging features of COQ8A‐ataxia, possibly indicating underlying metabolic changes related to COQ8A‐dysfunction. T2‐hyperintense dentate nuclei have also recently been found in another nuclear‐encoded mitochondrial recessive ataxia, SPG7.^28^ As both SPG7 and COQ8A are transmembrane proteins in the inner mitochondrial membrane, this imaging feature might point to similar underlying mitochondrial/metabolic pathway mechanisms.

### Natural Disease Progression of COQ8A‐Ataxia and Trial Design

Our cross‐sectional data indicate that disease progression in COQ8A is overall mild to moderate, with ~0.5 SARA points per year, and, thus, milder than disease progression in many other ARCAs like, for example, Friedreich’s ataxia (0.8 points per year) or Autosomal Recessive Spastic Ataxia of Charlevoix‐Saguenay (2.6 points per year).^29^, ^30^ This estimate from cross‐sectional data, which bears several methodological limitations (eg, recall bias of disease onset), was confirmed by preliminary prospective longitudinal data (SARA: 0.45/year). This progression estimate requires confirmation in future prospective natural history studies, which should overcome the limitations of our current study by (i) strictly defined, interindividually harmonized assessment intervals, (ii) larger patient cohorts, (iii) with sufficient statistical power to also assess the possible effects of covariates, like age of onset, disease duration, or genetic features, and, as in this study, (iv) leveraging the power of multivariate models. Nevertheless, this study already allows first preliminary sample size calculations for intervention trials in COQ8A disease. To detect a 50% reduction in annual SARA progression, at least 48 patients per trial arm may suffice. However, this preliminary estimate likely reflects the lowest limit of required subjects given that variability (ie, the SD) in the natural history cohort underlying this sample size calculation as well as the size of the cohort were relatively low.

### ***A Substantial Share of COQ8A Patients Seems to Respond to*** ***CoQ**_**10**_* ***Treatment***

Treatment with CoQ10 or its derivatives (eg, idebenone) has shown some beneficial effects in other recessive multisystemic ataxias (eg, *TXN2*),^31^ with at least mixed effects in Friedreich ataxia,^32^ primary CoQ10 deficiencies with neurological features (eg, *COQ2*, *COQ4*, or *COQ7*),^26^, ^33^, ^34^, ^35^ and respiratory dysfunction in Duchenne muscular dystrophy,^36^ and is already approved for Leber’s hereditary optic neuropathy.^37^ The efficacy of CoQ10 compounds is now even investigated in more common non‐ataxia neurodegenerative diseases (eg, mitochondrial Parkinson’s disease),^38^ thus highlighting the therapeutic potential of these compounds. Previous case series in COQ8A‐ataxia, often based on 1 to 2 patients and anecdotal experiences, have observed varying and partly inconsistent response to CoQ10.^4^, ^5^, ^6^ In this study, we present what we believe is the first analysis of CoQ10 treatment response in a larger cohort of COQ8A patients, also including quantitative longitudinal treatment data. Although our findings are limited by the small sample sizes and lack of a controlled study design, the complementary observations of (i) 43% responders by clinical report, (ii) improvement in quantitative longitudinal assessments, and (iii) clear individual “on/off”‐treatment effects (see Fig 6C) provide the strongest evidence to date that CoQ10 may stabilize or even improve ataxia severity in COQ8A‐ataxia. This prepares the rationale for a stringent randomized controlled trial required to confirm our preliminary observations. Such a well‐controlled trial is in particular need, as some degree of variation masking as “improvements” can be seen in the natural disease course of a small share of patients. Our qualitative treatment reports (and the delineation of the COQ8A phenotypic spectrum above) indicate that such future trials should also include outcome measures other than the SARA score for ataxia, allowing to capture possible effects of CoQ10 treatment beyond ataxia to include also tremor, dystonia, epilepsy, mental speed, or muscle weakness. Although a lower functional disease severity in responders suggests that treatment with CoQ10 may be more effective in less severe disease stages, no other clear predictor of treatment response was identified as of yet (eg, CoQ10 dose, disease duration, and mutation type). However, the patients reported here were treated with diverse (mostly over‐the‐counter) CoQ10 drugs, which might also in part explain the differences in treatment response. Because uptake of lipophilic CoQ10 in muscle and brain tissue is limited and requires high doses, future trials need to apply lipid‐soluble formulations to achieve relevant absorption and bioavailability of oral CoQ10.^39^, ^40^, ^41^ In conclusion, our findings motivate the planning of a randomized controlled trial in COQ8A‐ataxia as one of the first targeted treatment trials in ARCAs and guide the design thereof.

## Author Contributions

A.T., L.S., B.v.d.W., D.J.P., H.P., and M.S. contributed to the conception and design of the study. A.T., T.S., L.L., N.H.M., C.A.B., S.R., J.K., A.H., G.V., E.B., G.Z., A.D., S.M., F.T., A.M., J.B., P.d.J., W.d.R., M.B., S.D., C.G., A.N.B., H.H., S.H.K., B.B., U.G., T.K., R.H., B.v.d.W., L.B., C.R., C.E., M.K., F.M.S., M.A., R.P.M., T.H., F.D., D.J.P., and M.S. contributed to the acquisition and analysis of data. A.T., L.L., N.H.M., C.A.B., B.B., D.J.P., and M.S. contributed to drafting the text and preparing the figures.

## Potential Conflicts of Interest

The authors declared no conflict of interest.

## Supporting information


**Appendix S1:** Data sheet

**Appendix S2:** In silico predictions of all variants

**Appendix S3:** Characteristics of phenotypic cluster subgroups

**Appendix S4:** Clinico‐genetic associations with number of loss of function alleles

**Appendix S5:** Clinicogenetic associations with target motifs among missense variants

**Appendix S6:** Characteristics of imaging subgroups

**Appendix S7:** Characteristics of subgroups with data on disease progression

**Appendix S8:** Associations with effect of treatment with coenzyme Q10

**TABLE S1** COQ8A Variants Identified in this Study
