# Supplementary material for: Clinico‐Genetic, Imaging and Molecular Delineation of COQ8A ‐Ataxia: A Multicenter Study of 59 Patients
Source: Ann Neurol. 2020 Jun 10;88(2):251–63. doi: 10.1002/ana.25751 (PMC7877690; doi:10.1002/ana.25751)
Supplement: Supplementary file 2 — Appendix S2: In silico predictions of all variants [file ANA-88--s007.docx]

**Supplement 2 – In silico predictions of all variants**

| **Variant ID** | **cDNA Variant** | **Protein Change** | **Prediction** | **Short Prediction** | **Long Prediction** | **Secondary Structure Annotation** | **Region** |
| --- | --- | --- | --- | --- | --- | --- | --- |
| 1 | c.11T>A | I4K | missense_variant | Possible mitochondrial targeting issues | Not in the mature form of the protein, could disrupt mitochondrial targeting, typically a methionine in lower organisms | Predicted helix |  |
| 2 | c.124_125del | A42PfsTer21 | frameshift_variant | Early termination | early termination prior to the start of the mature protein, results in no mature protein | Predicted helix |  |
| 3 | c.148C>T | Q50Ter | stop_gained | Early termination | early termination prior to the start of the mature protein, results in no mature protein | Predicted helix |  |
| **4*** | c.238C>T | H80Y | missense_variant | Possible mitochondrial targeting issues | Unconserved histidine prior to the start of the mature protein, could impact mitochondrial targeting, not likely to impact protein function | No secondary structure prediction |  |
| 5 | c.248dup | H85AfsTer42 | frameshift_variant | Early termination | early termination prior to the start of the mature protein, results in no mature protein | No secondary structure prediction |  |
| 6 | c.447del | R150EfsTer59 | frameshift_variant | Early termination | Frameshift prior to the start of the mature protein, results in no mature protein | No secondary structure prediction |  |
| 7 | c.500_521delinsTTG | Q167LfsTer36 | frameshift_variant | Early termination | Frameshift 5 residues into the mature protein, results in dysfunctional protein | No secondary structure prediction |  |
| 8 | c.589-3C>G | - | splice_region_variant,intron_variant | Potential splicing change | Intron variation, could impact splicing |  |  |
| 9 | c.620C>T | P207L | missense_variant | Transmembrane helix disruption | Mutation in highly conserved region N-terminal of the transmembrane helix, unknown function of this region. Proline may act as a break between alpha helices so disruption could negatively impact the structure | Between predicted helices | Near TM domain |
| 10 | c.637C>T | R213W | missense_variant | Transmembrane helix disruption | R213 is directly adjacent to the transmembrane domain and is predicted to be a part of the same alpha helix. Mutation to a glycine could negatively impact helix formation or association with the membrane | Predicted transmembrane helix | Near TM Domain |
| 11 | c.637C>G | R213G | missense_variant | Transmembrane helix disruption | R213 is directly adjacent to the transmembrane domain and is predicted to be a part of the same alpha helix. Mutation to a glycine could negatively impact helix formation or association with the membrane | Predicted transmembrane helix | Near TM Domain |
| 12 | c.798_823dup | L275RfsTer16 | frameshift_variant | Early termination | Early frameshift and termination of the protein resulting in dysfunctional protein | GQalpha2 | N-terminal extension |
| 13 | c.802T>C | C268R | missense_variant | Steric and electrostatic clashes with adjacent residues | Shift in charge and size will likely introduce steric issues and negatively interact with adjacent hydrophobic residues | GQalpha1 | N-terminal extension |
| **14** | c.811C>T | R271C | missense_variant | Loss of electrostatic interactions | Loss of arginine bidentate interaction with V300 carbonyl and electrostatic interactions with the carbonyls on R301, A339, and S303 | GQalpha1 | N-terminal extension |
| 15 | c.815G>A | G272D | missense_variant | GQalpha1 helix/A-rich loop disruption | Introduction of a larger charged residue will introduce steric clashes with the carbonyl of S340 and the sidechains of I341 and C405. This will disrupt interactions with the GQalpha1 helix and the A-rich loop | Between GQalpha1 and GQalpha2 | N-terminal extension |
| 16 | c.815G>T | G272V | missense_variant | Introduction of steric clashes | Introduction of a larger residue will sterically clash with adjacent residues in the GQalpha helices and the A-rich loop | Between GQalpha1 and GQalpha2 | N-terminal extension |
| 17 | c.895C>T | R299W | missense_variant | Possible folding issues | Disordered in crystal structure and solvent exposed, may interfere with folding or solubility | GQalpha3 | N-terminal extension |
| **18** | c.901C>T | R301W | missense_variant | GQalpha3 helix disruption | May project into the substrate pocket or disrupt the GQalpha3 helix | GQalpha3 | N-terminal extension |
| 19 | c.910G>A | A304T | missense_variant | Steric clash with adjacent residues | Potential clashes with R271, F306, and Q343 | GQalpha3 | N-terminal extension |
| 20 | c.911C>T | A304V | missense_variant | Steric clash with adjacent residues | Potential clashes with R271, F306, and Q343 | GQalpha4 | N-terminal extension |
| **21*** | c.976G>A | D326N | missense_variant | No clear issues | Surface exposed asparatate. Mutation to asparagine shouldn't have major structural implications | Between GQalpha4 and Beta1alpha | N-terminal extension |
| 22** | c.993C>T | F331= | synonymous_variant | No change | No change in protein coding | Beta1alpha | PKL I |
| 23 | c.1009G>A | A337T | missense_variant | Steric clash with nucleotide and adjacent residues | Potential steric clashes with V334 and F336. Introduction of a larger residue will clash with bound nucleotide | Beta1beta (A-rich loop) | PKL I |
| **24** | c.1012G>A | A338T | missense_variant | Steric clash with adjacent residues | Potential steric clashes with the peptide backbone in the beta sheet, R271, A304, L275 and A304 | Beta1beta (A-rich loop) | PKL I |
| 25 | c.1013C>T | A338V | missense_variant | Steric clash with adjacent residues | Potential steric clashes with A304, L275, and backbone carbonyls | Beta1beta (A-rich loop) | PKL I |
| 26 | c.1015G>A | A339T | missense_variant | Steric clash with nucleotide and adjacent residues | Potential steric clashes with N492, K358 and multiple backbone carbonyls | Beta1beta (A-rich loop) | PKL I |
| 27 | c.1024G>T | G342W | missense_variant | Severe steric clashes | Massive steric clash, this residue resides in a location in phi-psi space that can only accommodate a glycine | beta2 | PKL I |
| 28 | c.1042C>T | R348Ter | stop_gained | Early termination | Termination prior to functional residues, leads to dysfunctional protein | Beta2 | PKL I |
| 29 | c.1081-1_1082dup | Q360_Y361insTer | stop_gained,splice_region_variant | Potential splicing change, early termination | Termination prior to functional residues, leads to dysfunctional protein | Beta3 | PKL II |
| 30 | c.1205T>C | L402P | missense_variant | GQalpha6 helix disruption | Mutation will disrupt GQalpha6 helix formation | GQalpha6 | N-lobe insert |
| 31 | c.1228C>T | R410Ter | stop_gained | Early termination | Termination prior to functional residues, leads to dysfunctional protein | alphaC | PKL III |
| 32 | c.1332_1336dup | E446AfsTer33 | frameshift_variant | Early termination | Termination prior to functional residues, leads to dysfunctional protein | Beta5 | PKL V |
| 33 | c.1334_1335del | T445RfsTer52 | frameshift_variant | Early termination | Termination prior to functional residues, leads to dysfunctional protein | Beta5 | PKL V |
| 34 | c.1358del | L453RfsTer24 | frameshift_variant | Early termination | Termination prior to functional residues, leads to dysfunctional protein | Between Beta5 and AlphaE | PKL V |
| 35 | c.1399-3_1408del | - | splice_acceptor_variant,  coding_sequence_variant,  intron_variant | Splice variant | Splice variant |  |  |
| 36 | c.1440delinsTT | E481Ter | frameshift_variant | Early termination | Termination prior to functional residues, leads to dysfunctional protein | alphaE | PKL Via |
| **37** | c.1460C>G | T487R | missense_variant | Potential steric and electrostatic clash | Steric clashes due to the introduction of a large polar residue which could impact folding. There is also a potential gain of salt bridges to D468 and E401 and repulsion with R512 | between Beta6 and Beta7 | PKL Vib |
| 38 | c.1523T>C | F508S | missense_variant | Loss of hydrophobic interactions | Loss of hydrophobic interactions with V430 and F418. Serine may be desolvated and somewhat inaccessible | between Beta8 and Beta9 | PKL VII |
| 39 | c.1532C>T | T511M | missense_variant | Steric clash with adjacent residues | Potential steric clash with F508, F418, and F484 | Beta9 | PKL VII |
| 40 | c.1534C>T | R512W | missense_variant | Loss of salt bridge and introduction of electrostatic clash | Loss of salt bridge with E401. Mutation may also introduce steric clash and put tryptophan against backbone carbonyl groups | Beta9 | PKL VII |
| 41 | c.1645G>A | G549S | missense_variant | Steric clash | Due to the location in phi-psi space any residue but glycine will introduce significant steric clash with adjacent residues | Between Calpha2 and Calpha3 | C-lobe insert |
| 42 | c.1651G>A | E551K | missense_variant | Electrostatic clash | Mutation will result in electrostatic clash with R598 | Between Calpha2 and Calpha3 | C-lobe insert |
| 43 | c.1665G>A | M555I | missense_variant | Steric clash with V552 backbone | Introduction of a beta-branched residue may cause clash with the carbonyl on V552 | Calpha3 | C-lobe insert |
| 44 | c.1702G>T | E568Ter | stop_gained | Early termination | Removes 79 C-terminal residues, this region of the protein is generally uncharacterized but removal is likely to disrupt function | Calpha3 | C-lobe insert |
| 45 | c.1750_1752del | T584del | inframe_deletion | Loss of interaction with adjacent residues | May lose interactions with S621 and adjacent residues | Calpha4 | C-lobe insert |
| 46 | c.1813dup | E605GfsTer125 | frameshift_variant | Early termination | Frameshift 42 residues prior to C-terminus, this region hasn't been well characterized, but could disrupt function | alphaF | PKL IX |
| 47* | c.1821C>T | Y607= | synonymous_variant | No change | No change in protein coding | alphaF | PKL IX |
| 48 | c.1844G>A | G615D | missense_variant | Steric clash with adjacent carbonyl oxygens | Introduction of a large charged residue could alter helix formation and clash with surrounding carbonyl oxygen residues | alphaF | PKL IX |
