# Supplementary material for: Clinico‐Genetic, Imaging and Molecular Delineation of COQ8A ‐Ataxia: A Multicenter Study of 59 Patients
Source: Ann Neurol. 2020 Jun 10;88(2):251–63. doi: 10.1002/ana.25751 (PMC7877690; doi:10.1002/ana.25751)
Supplement: Supplementary file 3 — Appendix S3: Characteristics of phenotypic cluster subgroups [file ANA-88--s006.docx]

**Supplement 3 – Characteristics of phenotypic cluster subgroups**

|  | **Full Cohort (n≤59)** | **Cluster 1  (n≤14)** | **p-value vs. rest  of cohort** | **Cluster 2**  **(n≤13)** | **p-value vs. rest  of cohort** | **Cluster 3 (n≤9)** | **p-value vs. rest  of cohort** | **Cluster 4 (n≤12)** | **p-value vs. rest  of cohort** | **Cluster 5 (n≤11)** | **p-value vs. rest  of cohort** | **p-value between clusters** |
| --- | --- | --- | --- | --- | --- | --- | --- | --- | --- | --- | --- | --- |
| Age of onset  (years) | 8.9±9.2 | 8.0±6.6 | *0.679* | 4.5±5.7 | *0.051* | 9.8±8.3 | *0.259* | 8.0±6.8 | *0.655* | 15.4±14.6 | *0.051* | *0.063* |
| Disease duration  (years) | 25.8±16.6 | 29.6±19.9 | *0.332* | 20.5±14.4 | *0.218* | 25.4±11.2 | *0.568* | 28.5±18.0 | *0.457* | 24.1±17.4 | *0.976* | *0.682* |
| Disease severity (SDFS) | 2 [2-3] | 3.0 [2-4] | *0.210* | 2.0 [2-4] | *0.815* | 2 [2-3] | *0.525* | 2 [2-3] | *0.650* | 2 [2-4] | *0.777* | *0.727* |
| Disease severity (SARA) | 11.8±4.6 | 14.2±4.6 | *0.167* | 12.9±4.4 | *0.452* | 12.0±4.1 | *0.830* | 10.6±4.4 | *0.723* | 10.0±5.4 | *0.160* | *0.454* |
| Genetics  (biallelic LOF) | 29% | 54% | ***0.036*** | 23% | *0.430* | 8% | *0.097* | 27% | *1.000* | 25% | *1.000* | *0.211* |

Descriptive statistics (mean ± standard deviation, or median [IQR]) of patient subgroups in phenotypic clusters. For each subgroup (cluster 1: “ataxia simplex”, cluster 2: cognitive impairment, cluster 3: epilepsy, cluster 4: myoclonus or dystonia, cluster 5: exercise intolerance), statistical comparison with the rest of the cohort was made with t-tests for numeric data, the Mann-Whitney U test for the SDFS, and Fisher’s exact test for proportions. Differences between clusters were analyzed with the Kruskal-Wallis test for the SDFS, and analysis of variance (ANOVA) for other numeric data, and Fisher’s exact test for proportions. There was no significant difference between phenotypic clusters, and each cluster was statistically representative of the full cohort, except a significantly higher prevalence of biallelic loss of function (LOF) mutations in patients with “ataxia simplex”.
