# Supplementary material for: Clinico‐Genetic, Imaging and Molecular Delineation of COQ8A ‐Ataxia: A Multicenter Study of 59 Patients
Source: Ann Neurol. 2020 Jun 10;88(2):251–63. doi: 10.1002/ana.25751 (PMC7877690; doi:10.1002/ana.25751)
Supplement: Supplementary file 4 — Appendix S4: Clinico‐genetic associations with number of loss of function alleles [file ANA-88--s008.docx]

**Supplement 4 – Clinico-genetic associations with number of loss of function alleles**

|  | **0 LOF alleles (n≤28)** | **1 LOF allele (n≤14)** | **2 LOF alleles (n≤17)** | **p-value of Fisher’s exact test** |
| --- | --- | --- | --- | --- |
| Cluster 1  (“Ataxia simplex”) | 5/23 (18%) | 1/14 (7%) | 8/17 (47%) | ***0.029*** |
| Epilepsy | 13/28 (46%) | 4/14 (29%) | 2/17 (12%) | ***0.046*** |
| Myoclonus | 12/27 (44%) | 2/14 (14%) | 2/15 (13%) | ***0.041*** |
| Dystonia | 8/28 (29%) | 5/14 (36%) | 3/15 (20%) | *0.637* |
| Head tremor | 8/26 (31%) | 3/13 (23%) | 1/15 (7%) | *0.207* |
| Bradykinesia | 6/27 (22%) | 1/14 (7%) | 2/15 (13%) | *0.524* |
| Slow saccades | 3/25 (12%) | 2/14 (14%) | 2/17 (12%) | *1.000* |
| Cognitive impairment | 12/28 (43%) | 7/14 (50%) | 7/17 (41%) | *0.889* |
| Intellectual disability | 8/28 (29%) | 2/14 (14%) | 3/17 (17%) | *0.555* |
| Developmental delay | 10/24 (42%) | 9/14 (64%) | 4/15 (27%) | *0.134* |
| Neuropsychiatric features | 5/26 (19%) | 2/14 (14%) | 5/16 (31%) | *0.494* |
| Exercise intolerance | 6/24 (25%) | 3/13 (23%) | 2/13 (15%) | *0.907* |
| Impaired vibration sense | 3/17 (18%) | 0/12 (0%) | 0/12 (0%) | *0.483* |
| Pyramidal signs | 3/28 (11%) | 1/14 (7%) | 3/15 (20%) | *0.662* |
| Migraine | 2/25 (8%) | 3/13 (23%) | 1/14 (7%) | *0.456* |
| Impaired strength | 4/25 (16%) | 1/13 (8%) | 1/13 (8%) | *0.746* |
| Bladder dysfunction | 5/27 (19%) | 1/14 (7%) | 0/14 (0%) | *0.290* |
| Hearing loss | 2/26 (8%) | 2/14 (14%) | 2/15 (13%) | *0.637* |

Clinico-genetic association between the number of loss of function (LOF) alleles and (i) the prevalence of cluster 1 with predominant ataxia (“ataxia simplex”), and (ii) the prevalence of frequent (>10% of patients) non-ataxia features. Numerator indicates number of affected patients, denominator indicates number of patients with available data on corresponding feature, the percentage affected is shown in brackets. “Ataxia simplex” (cluster 1 from cluster analysis) is more frequent in patients with biallelic LOF mutations. Consistent with this, epilepsy and myoclonus are more frequent in patients without LOF mutations, i.e. patients with missense variants.
