# Supplementary material for: Clinico‐Genetic, Imaging and Molecular Delineation of COQ8A ‐Ataxia: A Multicenter Study of 59 Patients
Source: Ann Neurol. 2020 Jun 10;88(2):251–63. doi: 10.1002/ana.25751 (PMC7877690; doi:10.1002/ana.25751)
Supplement: Supplementary file 5 — Appendix S5: Clinicogenetic associations with target motifs among missense variants [file ANA-88--s001.docx]

**Supplement 5 – Clinico-genetic associations with target motifs among missense variants**

|  | **AAAS motif (n≤7)** | **Non-AAAS**  **(n≤35)** | **p-value, Fisher’s exact test** | **GQα1 motif**  **(n≤10)** | **Non-GQα1**  **(n≤32)** | **p-value, Fisher’s exact test** | **GQα3 motif**  **(n≤14)** | **Non-GQα3**  **(n≤28)** | **p-value, Fisher’s exact test** |
| --- | --- | --- | --- | --- | --- | --- | --- | --- | --- |
| Cluster 1 (“Ataxia simplex”) | 3/7 (43%) | 3/35 (9%) | ***0.048*** | 0/10 (0%) | 6/32 (19%) | *0.308* | 1/14 (7%) | 5/28 (18%) | *0.645* |
| Epilepsy | 0/7 (0%) | 17/35 (49%) | ***0.030*** | 8/10 (80%) | 9/32 (28%) | ***0.008*** | 8/14 (57%) | 9/28 (32%) | *0.184* |
| Myoclonus | 1/7 (14%) | 13/34 (38%) | *0.389* | 5/9 (56%) | 9/32 (28%) | *0.231* | 4/13 (31%) | 10/28 (36%) | *1.000* |
| Dystonia | 1/7 (14%) | 12/35 (34%) | *0.405* | 4/10 (40%) | 9/32 (28%) | *0.697* | 5/14 (29%) | 8/28 (29%) | *0.729* |
| Head tremor | 2/7 (29%) | 9/32 (28%) | *1.000* | 2/8 (25%) | 9/31 (29%) | *1.000* | 5/14 (29%) | 6/25 (24%) | *0.478* |
| Bradykinesia | 3/7 (43%) | 4/34 (12%) | *0.082* | 1/9 (11%) | 6/32 (28%) | *1.000* | 3/14 (21%) | 4/27 (15%) | *0.673* |
| Slow saccades | 0/7 (0%) | 5/32 (16%) | *0.563* | 2/7 (29%) | 3/32 (9%) | *0.213* | 2/14 (14%) | 3/25 (12%) | *1.000* |
| Cognitive impairment | 4/7 (57%) | 15/35 (43%) | *0.682* | 5/10 (50%) | 14/32 (44%) | *1.000* | 8/14 (57%) | 11/28 (39%) | *0.335* |
| Intellectual Disability | 0/7 (0%) | 10/35 (29%) | *0.168* | 5/10 (50%) | 5/32 (16%) | ***0.040*** | 2/14 (14%) | 8/28 (29%) | *0.451* |
| Developmental delay | 2/6 (33%) | 17/32 (53%) | *0.660* | 4/9 (44%) | 15/29 (52%) | *1.000* | 7/14 (50%) | 12/24 (50%) | *1.000* |
| Neuropsychiatric features | 1/7 (14%) | 6/33 (18%) | *1.000* | 1/9 (11%) | 6/31 (19%) | *1.000* | 3/14 (21%) | 4/26 (15%) | *0.679* |
| Exercise intolerance | 3/7 (29%) | 6/30 (20%) | *0.327* | 4/8 (50%) | 5/29 (17%) | *0.078* | 2/12 (17%) | 7/25 (28%) | *0.687* |
| Impaired vibration sense | 1/5 (20%) | 2/24 (8%) | *0.446* | 1/4 (25%) | 2/25 (8%) | *0.371* | 0/14 (0%) | 4/28 (14%) | *0.283* |
| Pyramidal signs | 0/7 (0%) | 4/35 (11%) | *1.000* | 4/10 (40%) | 0/32 (0%) | ***0.002*** | 0/14 (0%) | 4/28 (14%) | *0.283* |
| Migraine | 1/7 (14%) | 4/31 (13%) | *1.000* | 2/8 (25%) | 3/30 (10%) | *0.279* | 2/11 (18%) | 3/27 (11%) | *0.615* |
| Impaired strength | 1/7 (14%) | 4/31 (13%) | *1.000* | 2/6 (33%) | 3/32 (9%) | *0.169* | 0/14 (0%) | 5/24 (21%) | *0.137* |
| Bladder dysfunction | 2/7 (29%) | 4/34 (13%) | *0.268* | 0/9 (0%) | 6/32 (19%) | *0.309* | 2/14 (14%) | 4/27 (15%) | *1.000* |
| Hearing loss | 1/7 (14%) | 3/33 (9%) | *0.552* | 0/8 (0%) | 4/32 (13%) | *0.566* | 2/13 (15%) | 2/27 (7%) | *0.584* |

Clinicogenetic association between affected target motif and phenotype among patients with missense variants, including (i) association with the prevalence of cluster 1 with predominant ataxia (“ataxia simplex”), and (ii) association with the prevalence of frequent (> 10% of patients) non-ataxia features. Numerator indicates number of affected patients, denominator indicates number of patients with available data on corresponding feature, the percentage affected is shown in brackets.
