# Supplementary material for: Clinico‐Genetic, Imaging and Molecular Delineation of COQ8A ‐Ataxia: A Multicenter Study of 59 Patients
Source: Ann Neurol. 2020 Jun 10;88(2):251–63. doi: 10.1002/ana.25751 (PMC7877690; doi:10.1002/ana.25751)
Supplement: Supplementary file 6 — Appendix S6: Characteristics of imaging subgroups [file ANA-88--s002.docx]

**Supplement 6 – Characteristics of imaging subgroups**

|  | **Full Cohort (n≤59)** | **MRI by report (n≤50)** | **p-value vs. rest  of cohort** | **MRI by review**  **(n≤18)** | **p-value vs. rest  of cohort** | **DTI analysis (n≤3)** | **p-value vs. rest  of cohort** |
| --- | --- | --- | --- | --- | --- | --- | --- |
| Age of onset  (years) | 8.9±9.2 | 8.4±8.0 | *0.288* | 7.1±5.5 | *0.310* | 6.0±5.0 | *0.580* |
| Disease duration  (years) | 25.8±16.6 | 27.2±17.1 | *0.103* | 20.2±17.8 | *0.097* | 12.0±5.3 | *0.142* |
| Disease severity (SDFS) | 2 [2-3] | 2 [2-3] | *0.423* | 2 [2-3] | *0.772* | 2 [-] | *0.844* |
| Disease severity (SARA) | 11.8±4.6 | 12.0±4.7 | *0.309* | 11.3±4.7 | *0.685* | 9.0±2.6 | *0.282* |
| Genetics  (biallelic LOF) | 29% | 38% | *1.000* | 40% | *1.000* | 50% | *1.000* |
| Phenotype  (“ataxia simplex”) | 24% | 24% | *1.000* | 17% | *0.516* | 0% | *1.000* |

Descriptive statistics (mean ± standard deviation, or median [IQR]) of patient subgroups with different analysis of imaging data. For each subgroup (analysis of MRI by report alone, centralized review of MRI data, DTI analysis), statistical comparison with the rest of the cohort was made with t-tests for numeric data, the Mann-Whitney U test for the SDFS, and Fisher’s exact test for proportions. Each subgroup was statistically representative of the full cohort with respect to the analyzed features.
