# Supplementary material for: Clinico‐Genetic, Imaging and Molecular Delineation of COQ8A ‐Ataxia: A Multicenter Study of 59 Patients
Source: Ann Neurol. 2020 Jun 10;88(2):251–63. doi: 10.1002/ana.25751 (PMC7877690; doi:10.1002/ana.25751)
Supplement: Supplementary file 7 — Appendix S7: Characteristics of subgroups with data on disease progression [file ANA-88--s003.docx]

**Supplement 7 – Characteristics of subgroups with data on disease progression**

|  | **Full Cohort (n≤59)** | **Cross-sectional (n≤34)** | **p-value vs. rest  of cohort** | **Longitudinal drug-naive**  **(n≤7)** | **p-value vs. rest  of cohort** | **Longitudinal on CoQ_10_ (n≤11)** | **p-value vs. rest  of cohort** | **p-value vs. drug-naive** |
| --- | --- | --- | --- | --- | --- | --- | --- | --- |
| Age of onset  (years) | 8.9±9.2 | 9.3±8.5 | *0.677* | 7.6±4.7 | *0.688* | 9.0±6.6 | *0.967* | *0.453* |
| Disease duration  (years) | 25.8±16.6 | 25.2±16.6 | *0.763* | 27.3±19.0 | *0.802* | 20.9±13.3 | *0.283* | *0.497* |
| Disease severity (SDFS) | 2 [2-3] | 2 [2-3] | *0.870* | 2 [2-3] | *0.872* | 2 [2-3] | *0.525* | *0.524* |
| Disease severity (SARA) | 11.8±4.6 | 11.8±4.6 | *not applicable* | 12.6±4.6 | *0.619* | 11.1±3.1 | *0.531* | *0.429* |
| Genetics  (biallelic LOF) | 29% | 36% | *1.000* | 25% | *1.000* | 50% | *0.619* | *1.000* |
| Phenotype  (“ataxia simplex”) | 24% | 18% | *0.229* | 29% | *0.666* | 27% | *0.712* | *1.000* |

Descriptive statistics (mean ± standard deviation, or median [IQR]) of patient subgroups with different data on disease progression. For each subgroup (cross-sectional SARA scores, longitudinal SARA scores in untreated patients, longitudinal SARA scores of patients treated with CoQ_10_), statistical comparison with the rest of the cohort and between drug-native and treated patients was made with t-tests for numeric data, the Mann-Whitney U test for the SDFS, and Fisher’s exact test for proportions. Each subgroup was statistically representative of the full cohort with respect to the analyzed features, and there was no difference between drug-naïve and treated patients with longitudinal SARA scores.
