# Supplementary material for: Clinico‐Genetic, Imaging and Molecular Delineation of COQ8A ‐Ataxia: A Multicenter Study of 59 Patients
Source: Ann Neurol. 2020 Jun 10;88(2):251–63. doi: 10.1002/ana.25751 (PMC7877690; doi:10.1002/ana.25751)
Supplement: Supplementary file 8 — Appendix S8: Associations with effect of treatment with coenzyme Q10 [file ANA-88--s009.docx]

**Supplement 8 –Associations with effect of treatment with coenzyme Q10**

|  | **Non-Responders** | **N** | **Responders** | **N** | **p-value of comparison** |
| --- | --- | --- | --- | --- | --- |
| CoQ_10_ dose (mg/kg/day) | 12.5±9.7 | 14 | 9.2±5.8 | *13* | *0.292* |
| Age at treatment (years) | 20.5±14.0 | 4 | 30.6±16.9 | *9* | *0.322* |
| Disease duration at treatment (years) | 9.8±4.6 | 4 | 22.6±12.3 | *9* | *0.074* |
| Age at last examination (years) | 29.3±18.4 | 14 | 38.0±18.9 | *13* | *0.237* |
| Age of onset (years) | 11.3±12.0 | 15 | 11.3±11.3 | *13* | *0.995* |
| Disease duration (years) | 17.4±12.2 | 14 | 26.7±16.5 | *13* | *0.105* |
| Disease severity (SDFS) | 3 [2-4] | 14 | 2 [2-2] | *13* | ***0.012*** |
| Disease severity (SARA) | 11.9±2.8 | 7 | 10.5±3.4 | *9* | *0.403* |
| Genetics (biallelic LOF) | 5/11 (46%) |  | 4/8 (50%) |  | *1.000* |
| Cluster 1 (“Ataxia simplex”) | 4/15 (27%) |  | 2/13 (15%) |  | *0.655* |
| Epilepsy | 8/15 (53%) |  | 2/13 (15%) |  | *0.055* |
| Myoclonus | 5/13 (39%) |  | 3/12 (25%) |  | *0.673* |
| Dystonia | 3/13 (23%) |  | 5/13 (39%) |  | *0.673* |
| Cognitive impairment | 10/15 (67%) |  | 5/13 (39%) |  | *0.255* |
| Exercise intolerance | 3/11 (27%) |  | 4/12 (33%) |  | *1.000* |
| Cerebral atrophy on MRI | 2/5 (40%) |  | 1/14 (7%) |  | *1.000* |
| Mitochondrial abnormality in biopsy | 3/10 (30%) |  | 3/4 (75%) |  | *0.245* |
| CoQ_10_ deficiency in muscle | 5/5 (100%) |  | 2/4 (50%) |  | *0.167* |

Association of CoQ10 treatment effect with CoQ10 treatment, demographics, clinical phenotype, imaging features and laboratory findings. Numeric data are shown as median [IQR], or mean ± standard deviation, and corresponding comparisons were made using Mann-Whitney U test for the SDFS, and t-tests for all other data. In proportional data, numerators indicate number of affected patients, denominators indicate number of patients with available data on corresponding feature, the percentage affected is shown in brackets. Treatment responders had lower functional disease severity as indicated by the Spinocerebellar Degeneration Functional Score (SDFS).
