# Supplementary material for: Clinico‐Genetic, Imaging and Molecular Delineation of COQ8A ‐Ataxia: A Multicenter Study of 59 Patients
Source: Ann Neurol. 2020 Jun 10;88(2):251–63. doi: 10.1002/ana.25751 (PMC7877690; doi:10.1002/ana.25751)
Supplement: Supplementary file 9 — TABLE S1 COQ8A Variants Identified in this Study [file ANA-88--s005.docx]

TABLE S1 COQ8A Variants Identified in this Study

| Var.ID | Family ID | cDNA change | Protein change | Pred. effect | MAF gnomAD | CADD score | SIFT | Poly phen2 | fathmm MKL | HGMD /publ. |
| --- | --- | --- | --- | --- | --- | --- | --- | --- | --- | --- |
| 1 | 44 | c.11T>A | I4K | mis | 0.0001 | 22.70 | 0 | 0.02 | 0.94 | Yes |
| 2 | 8 | c.124_125del | A42PfsTer21 | fs | - |  | - | - | - | No |
| 3 | 8 | c.148C>T | Q50Ter | stop | - | 37.00 | - | - | 0.98 | Yes |
| 4[^a^](#TF1) | 48 | c.238C>T | H80Y | mis | 0.0064 | 15.81 | 0.03 | 0.04 | 0.87 | Yes |
| 5 | 12, 37 | c.248dup | H85AfsTer42 | fs | - | - | - | - | - | No |
| 6 | 1 | c.447del | R150EfsTer59 | fs | - | - | - | - | - | No |
| 7 | 16, 35 | c.500_521 delinsTTG | Q167LfsTer36 | fs | - | - | - | - | - | Yes |
| 8 | 19, 21, 32 | c.589-3C>G | - | can | 0.0001 | 13.75 | - | - | 0.40 | Yes |
| 9 | 36 | c.620C>T | P207L | mis | - | 26.90 | 0 | 1.00 | 0.97 | No |
| 10 | 33 | c.637C>T | R213W | mis | 0.0001 | 32.00 | 0 | 1.00 | 0.83 | Yes |
| 11 | 43 | c.637C>G | R213G | mis | - | 27.50 | 0 | 1.00 | 0.86 | No |
| 12 | 38 | c.798_823dup | L275RfsTer16 | fs | - | - | - | - | - | No |
| 13 | 39 | c.802T>C | C268R | mis | - | 25.50 | 0 | 0.47 | 0.96 | No |
| 14 | 5, 14, 18, 31 | c.811C>T | R271C | mis | 0.0001 | 27.20 | 0 | 1.00 | 0.93 | Yes |
| 15 | 34 | c.815G>A | G272D | mis | - | 26.00 | 0 | 1.00 | 0.98 | Yes |
| 16 | 33 | c.815G>T | G272V | mis | - | 25.90 | 0 | 1.00 | 0.99 | Yes |
| 17 | 6, 17, 29 | c.895C>T | R299W | mis | 0.0001 | 32.00 | 0 | 0.97 | 0.91 | Yes |
| 18 | 2, 21, 22, 24, 41 | c.901C>T | R301W | mis | 0.0001 | 23.20 | 0 | 0.99 | 0.47 | Yes |
| 19 | 14 | c.910G>A | A304T | mis | 0.0001 | 25.10 | 0 | 0.97 | 0.97 | Yes |
| 20 | 4, 7 | c.911C>T | A304V | mis | 0.0001 | 25.20 | 0 | 0.99 | 0.96 | Yes |
| 21[^a^](#TF1) | 49 | c.976G>A | D326N | mis | 0.0001 | 23.40 | 0.11 | 0.10 | 0.92 | No |
| 22[^b^](#TF2) | 51 | c.993C>T | F331= | crypt | 0.0159 | 8.59 | - | - | 0.09 | Yes |
| 23 | 10 | c.1009G>A | A337T | mis | 0.0001 | 27.00 | 0 | 1.00 | 0.97 | No |
| 24 | 1, 13 | c.1012G>A | A338T | mis | 0.0001 | 25.30 | 0 | 1.00 | 0.97 | No |
| 25 | 11 | c.1013C>T | A338V | mis | 0.0001 | 25.00 | 0 | 1.00 | 0.96 | No |
| 26 | 9 | c.1015G>A | A339T | mis | 0.0001 | 25.30 | 0 | 1.00 | 0.97 | Yes |
| 27 | 42 | c.1024G>T | G342W | mis | - | 26.70 | 0 | 1.00 | 0.97 | No |
| 28 | 16, 20, 23 | c.1042C>T | R348Ter | stop | 0.0001 | 41.00 | - | - | 0.84 | Yes |
| 29 | 9, 30 | c.1081-1_ 1082dup | Q360_Y361 insTer | can | - | - | - | - | - | Yes |
| 30 | 38 | c.1205T>C | L402P | mis | 0.0001 | 29.60 | 0.01 | 1.00 | 0.97 | No |
| 31 | 29, 50 | c.1228C>T | R410Ter | stop | 0.0001 | 40.00 | - | - | 0.91 | Yes |
| 32 | 22 | c.1332_1336dup | E446AfsTer33 | fs | - | - | - | - |  | No |
| 33 | 48 | c.1334_1335del | T445RfsTer52 | fs | - | 34.00 | - | - | - | Yes |
| 34 | 6, 45, 46 | c.1358del | L453RfsTer24 | fs | - | 34.00 | - | - | - | Yes |
| 35 | 2 | c.1399-3_ 1408del | - | can | - | - | - | - | - | No |
| 36 | 15 | c.1440delinsTT | E481Ter | fs | - | - | - | - | - | No |
| 37 | 5, 18 | c.1460C>G | T487R | mis | - | 28.70 | 0 | 1.00 | 0.98 | Yes |
| 38 | 28 | c.1523T>C | F508S | mis | - | 32.00 | 0 | 1.00 | 0.99 | Yes |
| 39 | 3 | c.1532C>T | T511M | mis | 0.0001 | 24.50 | 0 | 0.94 | 0.97 | Yes |
| 40 | 44 | c.1534C>T | R512W | mis | 0.0001 | 23.50 | 0 | 1.00 | 0.39 | No |
| 41 | 51 | c.1645G>A | G549S | mis | 0.0001 | 26.40 | 0.01 | 0.99 | 0.98 | Yes |
| 42 | 17 | c.1651G>A | E551K | mis | 0.0001 | 32.00 | 0 | 1.00 | 0.98 | Yes |
| 43 | 26, 40, 41 | c.1665G>A | M555I | mis | 0.0004 | 28.90 | 0 | 0.93 | 0.98 | Yes |
| 44 | 25, 45, 46 | c.1702G>T | E568Ter | stop | 0.0001 | 54.00 | - | - | 0.98 | Yes |
| 45 | 3, 13 | c.1750_1752del | T584del | del | - | 22.70 | - | - | - | Yes |
| 46 | 34 | c.1813dup | E605GfsTer125 | fs | - | - | - | - | - | Yes |
| 47[^a^](#TF1) | 50 | c.1821C>T | Y607= | crypt | 0.0001 | 22.60 | - | - | 0.93 | No |
| 48 | 19, 27 | c.1844G>A | G615D | mis | - | 32.00 | 0 | 1.00 | 0.98 | Yes |

Genomic positions according to genome build GRCh37/hg19. DNA changes based on ENST00000366777.3. Protein changes according to ensemble protein ID ENSP00000355739.3. Predicted effects: can = canonical splice; crypt = cryptic splice; del = deletion; fs = frameshift; mis = missense; MAF = minor allele frequency.

^a^ Variant of unknown significance.

^b^ Likely not pathogenic.

CADD score = scaled Combined Annotation Dependent Depletion score; COQ8A = coenzyme Q8A; fathmm-MKL = functional analysis through hidden Markov Models; gnomAD = Genome Aggregation Database; HGMD = Human Gene Mutation Database; PolyPhen-2 = Polymorphism Phenotyping version 2; Pred. = predicted; SIFT = Sorting Intolerant From Tolerant score.
